# Choline Chloride-Based Deep Eutectic Solvents for Efficient Polyphenol Extraction from White Mulberry (*Morus alba*)

**DOI:** 10.3390/molecules31071193

**Published:** 2026-04-03

**Authors:** Kaja Gliha, Manja Kurečič, Drago Kočar, Mitja Kolar

**Affiliations:** 1 Faculty of Chemistry and Chemical Technology, University of Ljubljana, Večna Pot 113, 1000 Ljubljana, Slovenia; kaja.gliha@fkkt.uni-lj.si; 2 Faculty of Mechanical Engineering, University of Maribor, Smetanova ul. 17, 2000 Maribor, Slovenia; manja.kurecic@um.si

**Keywords:** white mulberry, deep eutectic solvents, polyphenolic compounds, ultrasound-assisted extraction, HPLC

## Abstract

The efficiency of six deep eutectic solvents (DESs) based on choline chloride (ChCl) and various hydrogen bond donors (HBDs) was evaluated against a traditional organic solvent for extracting polyphenolic bioactive compounds from three different white mulberry samples (*Morus alba*), including branches, leaves, and fruits. Ultrasound-assisted extraction was performed under selected conditions identified for ChCl/glycerol DES: a 1:2 molar ratio of hydrogen bond acceptor to HBD, 20% water added to the DES, a temperature of 80 °C, and an extraction time of 30 min, providing a set of standard parameters for comparing the efficiency of different DESs. Extraction efficiencies were assessed using a developed and validated HPLC method, as well as total phenolic content and total flavonoid content assays. Among the tested DESs, those composed of ChCl and polyalcohols as HBDs showed the best performance. For branch and leaf samples, the ChCl/glycerol DES was the most effective, while for fruit samples, the ChCl/ethylene glycol DES showed the highest efficiency. In most polyphenol extractions tested, at least one DES achieved extraction efficiencies comparable to or higher than those obtained with methanol, except for flavonoids, for which DES yields were often lower. Overall, the results indicate that using DESs represents a greener and more sustainable approach to extracting bioactive compounds from white mulberry.

## 1. Introduction

White mulberry (*Morus alba*), a member of the *Moraceae* family, is a tree native to China and parts of Central Asia, where it has been extensively cultivated for over 4500 years because its leaves serve as the primary food source for silkworm larvae [1]. It is also a rich natural source of bioactive compounds, including polyphenols, anthocyanins, alkaloids, flavonoids, and polysaccharides, which have a wide range of physiological and pharmacological effects [2,3]. Among the phytochemicals in white mulberry, polyphenols are the most abundant group. The main non-flavonoid polyphenols in mulberry are resveratrol, oxyresveratrol, chlorogenic acid, mulberroside A, and maclurin, while typical flavonoids include rutin, quercetin, and anthocyanins [2]. There is considerable evidence for the health benefits of phenols isolated from various parts of the mulberry tree, including leaves, fruits, stem bark, branches, roots, and root bark, with reported antioxidant, antidiabetic, anti-inflammatory, antihyperlipidemic, and cholesterol-lowering effects [2,4,5,6,7,8]. Due to the great potential of white mulberry, especially in the pharmaceutical and food industries, the extraction of polyphenolic compounds is a highly active area of research.

In the pursuit of sustainable green processes in chemistry, green extraction techniques have attracted significant attention as the scientific community increasingly seeks to develop more environmentally friendly methods for extracting bioactive compounds that reduce or eliminate the use of harmful organic solvents [9]. In this context, a new class of green solvents called deep eutectic solvents (DESs) was introduced, first reported in the literature by Abbott et al. in 2003 [10]. Compared to traditional organic solvents such as methanol and ethanol, DESs are biodegradable and less toxic, making them more environmentally friendly [11]. Additionally, the compounds used for their synthesis are readily available and the synthesis process is simple [12,13]. They are typically used in combination with green extraction techniques such as ultrasound-assisted extraction (UAE) and microwave-assisted extraction (MAE) [14]. Both techniques offer advantages over conventional extraction methods, including reduced extraction times, lower energy consumption, and higher extraction yields. However, UAE is often preferred for extracting bioactive compounds with DESs due to its milder operating conditions and the wider availability of equipment in analytical laboratories (e.g., ultrasonic cleaning bath) [2,12].

DES is a mixture of two or more organic or inorganic compounds that liquefy under optimal conditions (temperature and stirring time) to form a stable eutectic. The components of a DES are linked by hydrogen bonds, creating a supramolecular structure. One component acts as a hydrogen bond acceptor (HBA), while the other serves as a hydrogen bond donor (HBD) [12]. Based on the types of starting compounds used, DES can be classified into five groups (types I to V) [11]. Among these, type III DESs are most commonly used for extraction, with a quaternary ammonium salt—usually choline chloride (ChCl)—as the HBA, while typical HBDs include polyalcohols, alcohols, amides, sugars, and organic acids [11,12,15].

The extraction efficiency of bioactive compounds from plant materials depends on multiple parameters that must be carefully considered when selecting appropriate DESs and defining the extraction conditions [16]. The physicochemical properties of DESs play an important role and can be tailored to specific requirements by adjusting the choice of HBD and HBA, as well as the molar ratio between HBA and HBD [17,18]. DESs are typically characterized by relatively high densities and viscosities due to strong hydrogen bonding between their components [19]. Elevated viscosity especially adversely affects extraction efficiency by limiting effective contact between the solvent and target compounds and by reducing mass transfer diffusivity from the solid plant matrix into the solvent phase [15]. Increasing the temperature and adding water to DES decreases both viscosity and density, which improves extraction efficiency by allowing greater penetration of the solvents into the sample [14,20]. However, excessive water content may disrupt the intrinsic DES structure by breaking the hydrogen bonds between its components and simultaneously weakening the interactions between the bioactive compounds and the solvent [21], ultimately resulting in diminished extraction yield [15]. Optimal water content is typically reported to be in the range of 20–40% [12]. Density and viscosity are among the key parameters that determine the applicability of DESs; other important parameters include polarity, surface tension, and pH [22].

A limited number of studies have been published since 2018, when the first research appeared on the use of DESs as green solvents for extracting bioactive compounds from white mulberry. This initial study explored UAE of polyphenols from mulberry leaves using 12 different DESs, with the best results achieved using a DES composed of choline chloride and citric acid (2:1, 25% water) [23]. M. Z. Gao et al. also investigated polyphenol extraction from mulberry leaves, obtaining the highest yields with a ChCl/glycerol DES (1:2, 20% water) and MAE [24]. Other plant materials from white mulberry that have been studied include roots for oxyresveratrol extraction with UAE (best DES: ChCl/glycerol, 1:2) [25], fruits for anthocyanin extraction with UAE (best DES: ChCl/lactic acid, 1:2, 20% water) [26], callus for stilbenoid extraction with UAE (best DES: ChCl/glycerol, 1:2, 30% water) [27], Fructus Mori for anthocyanin extraction with high-speed homogenization and cavitation-burst extraction (best DES: ChCl/citric acid/glucose, 1:1:1, 30% water) [28], branches for lignin extraction with classic solid–liquid extraction (best DES: lactic acid/ChCl, 2:1) [29], and root bark for mulberroside A extraction with UAE (best DES: malic acid/glucose, 1:3, 60% water) [30]. Recently, a novel method for simultaneous extraction and in situ separation of flavonoids and alkaloids from mulberry leaves using a pH-responsive DES/water system was also reported, with the optimal DES being hexanoic acid/2-methyl-2,4-pentanediol in a 1:1 molar ratio [31].

The purpose of this study was to evaluate the efficiency of six different DESs as green solvents for extracting polyphenol bioactive compounds using UAE, compared to methanol as a conventional organic solvent, in three morphologically distinct mulberry samples: branches, leaves, and fruits. Extraction parameters were determined through single-factor experiments using a single DES (ChCl/glycerol) to identify suitable conditions for a meaningful comparison of DES efficiency. A high-performance liquid chromatography (HPLC) method was developed and validated for quantifying five target analytes: gallic acid (GLA), caffeic acid (CFA), p-coumaric acid (pCA), rutin (RU), and resveratrol (RES). Total phenolic content (TPC) and total flavonoid content (TFC) were also evaluated. In addition, the prepared DESs were physicochemically characterized by measuring their densities and viscosities to better understand their effect on DES extraction efficiency. By comparing the extraction efficiency of DESs across three different parts of a plant within a single study, this work provides a systematic evaluation of DES performance for white mulberry that, to our knowledge, has not been previously reported.

## 2. Results and Discussion

### 2.1. Physicochemical Properties of DESs

#### 2.1.1. Density

**Figure 1:**
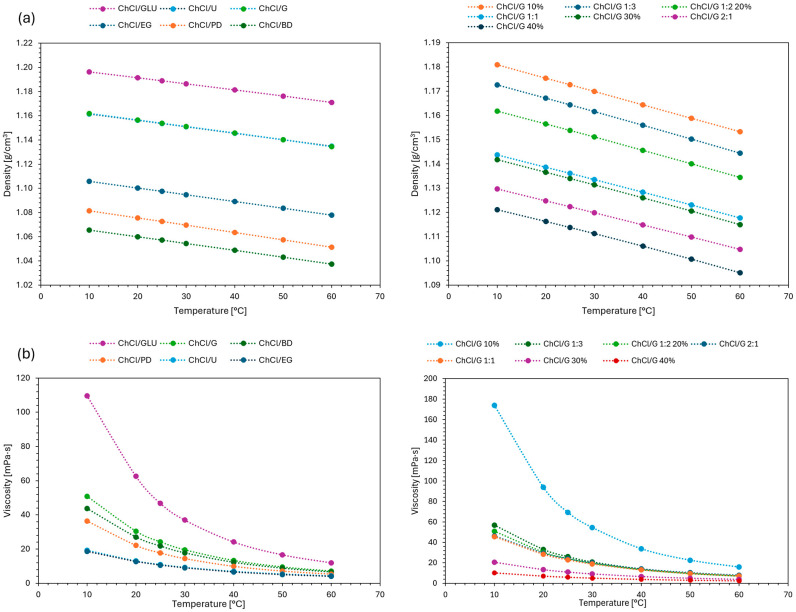
(**a**) Densities and (**b**) viscosities of deep eutectic solvents (DESs) as a function of temperature. Abbreviations: ChCl/G, choline chloride/glycerol; ChCl/EG, choline chloride/ethylene glycol; ChCl/PD, choline chloride/1,3-propanediol; ChCl/BD, choline chloride/1,4-butanediol; ChCl/U, choline chloride/urea; ChCl/GLU, choline chloride/glucose/water (2:1:1). Unless otherwise stated, DESs were prepared at a molar ratio of 1:2 with 20% (*w*/*w*) added water.

The results in Figure 1 (and Table S1) show that temperature, type of HBD, HBA/HBD molar ratio, and water content all affect the density of DESs. Density decreased almost linearly with increasing temperature, with similar slopes for all DESs. The highest densities were observed for ChCl/GLU, followed by ChCl/U and ChCl/G, which had similar values. After a notable gap, ChCl/EG and ChCl/PD followed, with ChCl/BD having the lowest density. This trend demonstrates the influence of HBD structure: a higher number of hydrogen bond-forming groups increases density, while longer carbon chains reduce intermolecular interactions due to greater steric hindrance, resulting in lower density [32]. Densities also increased as the HBA/HBD molar ratio changed from 2:1 to 1:3, but decreased with increasing water content. These changes in density reflect variations in the number and strength of intermolecular interactions within the DES [19,33].

#### 2.1.2. Viscosity

The results in Figure 1 (and Table S2) show that viscosity decreases exponentially as temperature increases. The highest viscosity was measured for ChCl/GLU, followed by ChCl/G, ChCl/BD, ChCl/PD, ChCl/U, and ChCl/EG. This sequence can be explained by the fact that viscosity increases with the length of the carbon chain, but the presence of functional groups that form hydrogen bonds can override this effect, as a higher number of intermolecular bonds also increases viscosity [34]. For example, glycerol in ChCl/G has a shorter carbon chain than 1,4-butanediol in ChCl/BD but contains more OH groups, resulting in slightly higher viscosity. Furthermore, viscosity increased with higher HBA/HBD molar ratios from 2:1 to 1:2 to 1:3, with values at a molar ratio of 1:1 being slightly lower than at 2:1. Viscosity also depends on the dilution of DESs, as a decrease was observed with increasing water content. Overall, viscosity increases with more HBD molecules due to stronger intermolecular interactions, but is reduced by higher water content, which disrupts them [19,33].

### 2.2. Optimization and Validation of HPLC Method

**Figure 2:**
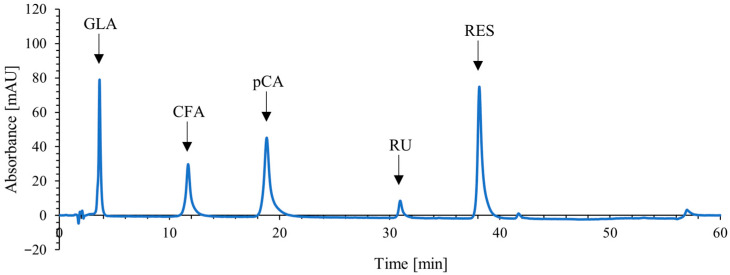
HPLC chromatogram of the standard solution containing 25 mg/L of each standard phenolic compound, with marked peaks for the targeted analytes: gallic acid (GLA), caffeic acid (CFA), p-coumaric acid (pCA), rutin (RU), and resveratrol (RES).

To optimize the chromatographic separation of five target polyphenolic bioactive compounds (GLA, CFA, p-CA, RU, RES) in a single chromatographic analysis run, a method was developed on an HPLC system with a diode array detector (DAD). Based on a literature review, gradient conditions were applied from the beginning of the method development [23,24]. Several experiments were performed to modify and adjust the separation gradient and conditions during subsequent development stages. All tests were conducted with both a standard solution containing pure phenolic target analytes and a preliminary extract, which was prepared as described in Section 3.4 from a mulberry branch sample using methanol as the solvent and an extraction temperature of 40 °C. Different mobile phase compositions were tested, consisting of acidified water (solvent A) and acetonitrile (solvent B). For solvent A, phosphoric acid and acetic acid at concentrations of 0.1%, 0.5%, and 1% were considered. The best separation of analytes was achieved using 0.1% phosphoric acid. Among the tested flow rates (1.0–1.3 mL/min), a flow rate of 1.1 mL/min was selected as optimal. Several injection volumes (10, 15, and 20 µL) were also tested, with the best results obtained by injecting 15 µL of solution into the column. Three wavelengths were considered for detection: 280, 320, and 360 nm. The wavelength of 280 nm was selected, as it allowed simultaneous detection of all target compounds while achieving the lowest noise levels. At the end of the optimization, the best separation of the target analytes was achieved with the following gradient conditions: 5–10% B (0–1 min), 10% B (1–3 min), 10–13% B (3–18 min), 13–18% B (18–27 min), 18–23% B (27–34 min), 23–35% B (34–44 min), 35–60% B (44–49 min), 60% B (49–54 min), 60–5% B (54–54.1 min), and 5% B (54.1–60 min). The total run time of the method was 60 min. All five analytes elute by the 38th minute, while the remaining time is used to flush more strongly retained compounds from the column and to re-establish the initial mobile phase composition. The chromatogram of the standard solution is shown in Figure 2; additional chromatograms of white mulberry extracts are provided in the Supplementary Materials.

To ensure that the developed method met the requirements for its intended analytical use, it was validated for linearity, intra-day and inter-day precision, limit of detection (LOD), limit of quantification (LOQ), accuracy, and selectivity. Good linearity was confirmed for all target analytes (R^2^ ≥ 0.9993) within the tested concentration range. Repeatability was evaluated as intra-day precision (system repeatability) and inter-day precision (intermediate precision). All five analytes showed consistently low relative standard deviation (%RSD) values for intra-day precision (≤1.5%) and inter-day precision (≤2.64%). Accuracy was assessed as the recovery rate, calculated by comparing known and measured concentrations of standard solutions. Recoveries for all analytes ranged from 97.4% to 102.6%. Selectivity was partially evaluated by testing all extraction solvents as potential sources of interference in the chromatographic analysis; no solvent-related peaks were detected in any chromatogram at the expected retention times of the five analytes. All validation parameters for the examined compounds, including mean retention times (*n* = 6), are presented in Table 1.

### 2.3. Establishment of Extraction Conditions

The extraction of bioactive compounds from plant material is influenced by several operational parameters. Based on single-factor experiments, the effects of the HBA/HBD molar ratio, added water content in DES, extraction temperature, and extraction time were evaluated. ChCl/G was used as the extraction solvent in all experiments because it had the shortest preparation time among the six DESs considered. Additionally, previous studies have demonstrated its high efficiency in extracting bioactive compounds from various parts of white mulberry [24,27]. Branches of white mulberry were used as the plant sample in all cases. Extraction efficiency was assessed by HPLC analysis, which served as the primary criterion for defining the most effective conditions and allowed quantification of CFA and RU, while pCA and RES remained below the LOQ or LOD, and GLA was not detected. TPC and TFC were also considered as secondary and tertiary criteria. These conditions were established to provide a suitable set of parameters for a consistent and meaningful comparison of different DESs.

#### 2.3.1. Effect of HBA/HBD Molar Ratio

**Figure 3:**
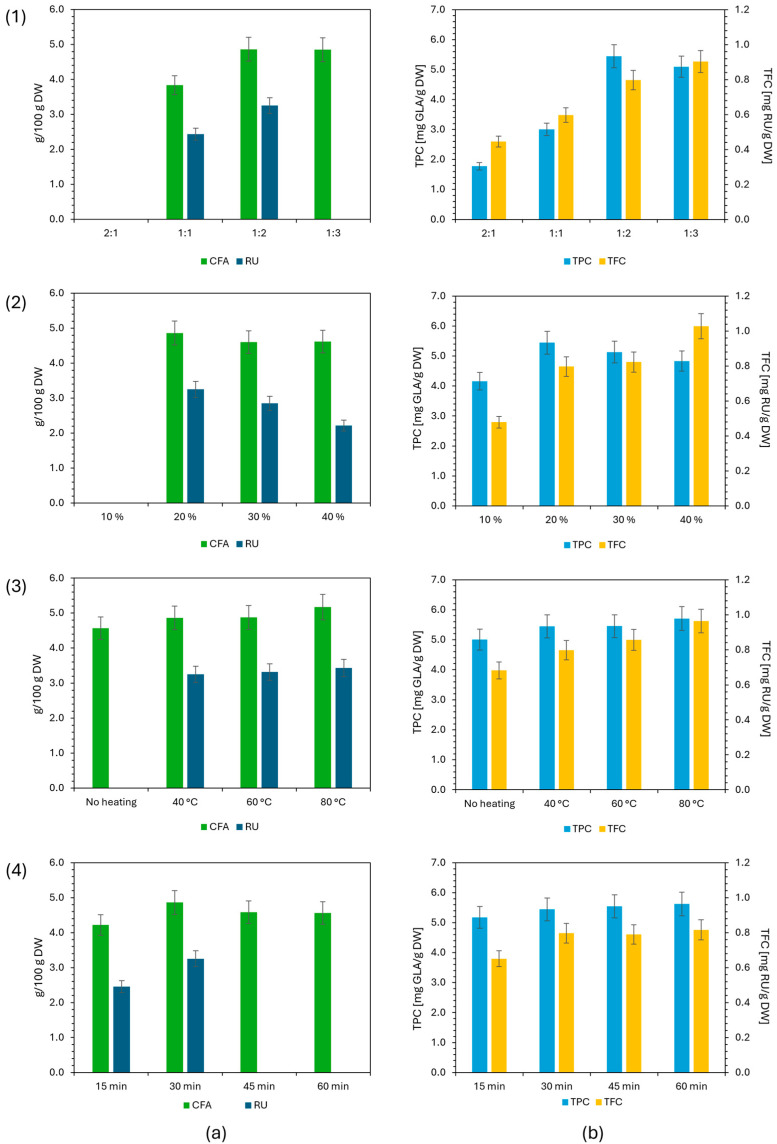
Effect of extraction parameters: (**1**) hydrogen bond acceptor/hydrogen bond donor (HBD/HBA) molar ratio, (**2**) added water content, (**3**) extraction temperature, and (**4**) extraction time on extraction efficiency determined by (**a**) the HPLC method and the analysis of (**b**) total phenolic content (TPC) and total flavonoid content (TFC). The results were expressed as mg of analyte or GLA or TFC per 100 g of dry weight (DW). The error bars represent an estimated %RSD of 7%, based on preliminary studies and prior experimental experience.

For a given type of DES, substantial differences in physicochemical properties may be observed when varying the molar ratio of HBA to HBD [22]. Therefore, molar ratios of 2:1, 1:1, 1:2, and 1:3 were evaluated to assess the influence of this extraction parameter on the final yield of the selected analytes (other extraction parameters were fixed at 20% added water, 40 °C, and 30 min). As shown in Figure 3, decreasing the molar ratio from 2:1 to 1:2 resulted in an increase in the extraction yield of CFA and RU, from values below the LOQ to the highest values obtained. This effect may be attributed to the increased glycerol (HBD) content, which reduced the viscosity from 14.05 mPa·s to 13.30 mPa·s at 40 °C, thereby facilitating diffusion and mass transfer within the system and improving extraction efficiency. When the molar ratio was further decreased from 1:2 to 1:3, the extraction yield of CFA did not change significantly and remained comparable between these two ratios, while the RU value fell below the LOQ. A similar trend was reported by Gao et al., who observed reduced extraction yields for phenolic analytes during MAE from white mulberry leaves with ChCl/G when the molar ratio was decreased from 1:2 to 1:5. The authors attributed this phenomenon to increased steric hindrance from the excess HBD, which weakened the interactions between the analytes and ChCl [24]. A similar dependence of extraction yield on the HBA/HBD molar ratio was observed for the TPC results, with an initial increase as the ratio decreased from 2:1 to 1:2, followed by a decrease at 1:3. In contrast, the TFC values increased continuously as the molar ratio decreased from 2:1 to 1:3. This difference could be attributed to polarity, which is also affected by the HBA/HBD ratio, as polarity increases with lower HBA and higher HBD content [35]. Based on all the discussed findings, the 1:2 ratio was selected as most suitable for further extractions, as both the primary (HPLC) and secondary (TPC) criteria showed a consistent trend supporting this ratio. All subsequent DESs were therefore prepared at this ratio, if they formed a stable eutectic under these conditions. This was not achievable with glucose as the HBD; thus, the corresponding DES could not be prepared at the designated molar ratio.

#### 2.3.2. Effect of Added Water Content in DES

DESs are generally characterized by high viscosities, which can hinder mass transfer during extraction and complicate subsequent experimental steps such as filtration and extract handling. The viscosity of DESs can be reduced by adding water. Accordingly, the influence of different water contents (10–40%, *w*/*w*) on the extraction efficiency of the target compounds was evaluated (other extraction parameters were fixed at HBA/HBD molar ratio 1:2, 40 °C, and 30 min). Higher water contents were not tested, as excess water disrupts the hydrogen bonding network between ChCl and HBD molecules, thereby eliminating the eutectic character of the mixture. As shown in Figure 3, the addition of 20% water resulted in the highest extraction efficiency for both quantified analytes, while further increases in water content led to decreased extraction yields. A similar trend was observed for the TPC results, whereas the TFC results indicated that a 40% water content was most favorable for the extraction of total flavonoids. A moderate amount of water reduces viscosity, with the biggest decrease step observed from 10 to 20% water from 33.70 mPa·s to 13.30 mPa·s at 40 °C, enhancing mass transfer and improving extraction performance. Conversely, increasing the water content beyond this optimum decreases extraction yield despite the reduction in viscosity, likely due to the increased polarity of the solvent [17], which is generally unfavorable for phenolic compounds [36]. The opposite effect was observed for flavonoids, likely due to differences in polarity resulting from variations in molecular structure among polyphenol subgroups [37]. Previous research showed that adding 25% water to ChCl/glycol DESs increased polarity to values close to those of pure water [21]. Based on these results, a water content of 20% was selected as most appropriate for subsequent extractions.

#### 2.3.3. Effect of Extraction Temperature

One of the key parameters influencing extraction efficiency is temperature, as increasing temperature reduces solvent viscosity, decreases density, and lowers surface tension [34]. Four extraction temperatures were evaluated: extraction without heating (ultrasonic bath heating switched off)—room temperature, 40 °C, 60 °C, and 80 °C (other extraction parameters were fixed at HBA/HBD molar ratio 1:2, 20% added water, and 30 min). This range covers the temperatures typically used for extracting bioactive compounds from plant materials using DES [15]. Higher temperatures were not tested, as excessive heat may cause degradation of target analytes [12]. The results shown in Figure 3 indicate that the lowest efficiencies in all cases occurred when extraction was performed without heating. This outcome is most likely due to the higher viscosity of the solvent, which imposes the greatest limitation on mass transfer, as viscosity significantly decreases from 24.22 mPa·s at 25 °C to 7.034 mPa·s at 60 °C. Similarly, a decrease in density could also be observed from 1.153793 g/cm^3^ at 25 °C to 1.134457 g/cm^3^ at 60 °C. As the extraction temperature increased, yields also improved, reaching their highest values at 80 °C, which was therefore selected as the most suitable temperature for further extractions. Based on the downward trend in viscosity and density with increasing temperature observed from 10 to 60 °C, as discussed in Section 2.4, it can be assumed that both values would be even lower at 80 °C [19,34]. Unfortunately, this could not be measured due to instrumentation limitations.

#### 2.3.4. Effect of Extraction Time

The final evaluated parameter of the extraction process was the extraction time. An optimal extraction duration typically increases analyte yield until equilibrium between the solid and liquid phases is reached. Reported extraction times for various DES systems generally range from 30 min to 4 h [15]. Shorter durations with high extraction efficiency are preferred, as prolonged exposure to temperatures higher than 50 °C may cause degradation of phenolic compounds, reducing extraction yield [38]. In this study, the maximum extraction time was limited to 1 h, and durations of 15, 30, 45, and 60 min were tested (other extraction parameters were fixed at HBA/HBD molar ratio 1:2, 20% added water, and 40 °C). As shown in Figure 3, the highest extraction efficiency for CFA and RU was achieved after 30 min. At longer extraction times, the yield for CFA slightly decreased, while RU fell below the LOQ, indicating partial degradation of these analytes. For TPC and TFC analysis, the extraction yield also increased at 30 min, while extended contact time did not significantly affect extraction efficiency, as the values remained comparable. Based on these results, an extraction time of 30 min was selected for subsequent experiments.

### 2.4. Evaluation of Extraction Efficiency of DESs

#### 2.4.1. Results of the HPLC Analysis

To assess the extraction efficiency of DESs using the developed chromatographic method, five polyphenolic compounds were selected: GLA, CFA, p-CA, RU, and RES. Results are presented in Table 2.

Four of the five target analytes were identified in all mulberry branch extracts, while CFA and RU were the only ones quantified. For GLA, no peak was detected at the corresponding retention time. For the extraction of CFA, the most efficient DESs were ChCl/G and ChCl/GLU, while all DESs except ChCl/PD and ChCl/BD showed efficiencies comparable to methanol. For RU, the most effective DES was also ChCl/G, although it achieved approximately 37% lower yield than methanol. For both quantified analytes, ChCl/BD was the least efficient extraction solvent. Considering the total yield, defined as the sum of all quantified analytes, the most effective DES was ChCl/G (9.1 mg/100 g DW), which was nevertheless less efficient than methanol (11.3 mg/100 g DW).

GLA and RES were identified in all mulberry leaf extracts, whereas only RU could be quantified. The highest yield of RU was obtained using ChCl/G, which was approximately 87% higher than that achieved with methanol. Yields higher than those with methanol were also achieved with three other DESs: ChCl/GLU, ChCl/U, and ChCl/EG. The least efficient extraction solvent was ChCl/BD.

In extracts of mulberry fruit samples, GLA, CFA, and RU were identified in all cases. CFA and RU were also quantified, while GLA could not be quantified due to insufficient separation. The most efficient DES for extracting CFA was ChCl/EG, which achieved an approximately 85% higher yield compared to methanol. Improved efficiency was also observed for all other tested DESs except ChCl/BD, whose performance was comparable to methanol. For RU, the most efficient DES was ChCl/PD, although its yield was about 16% lower than that of methanol. The least efficient extraction solvent in this case was ChCl/GLU. Considering total yield, the most effective solvent was ChCl/EG (43.5 mg/100 g DW), with ChCl/PD (41.7 mg/100 g DW) also outperforming methanol (39.0 mg/100 g DW).

It can be concluded that the efficiency of the tested DESs varied depending on the sample type, target analytes, and the composition of the different DESs. The efficiency order of DESs for RU was consistent in both branch and leaf extracts, showing a similar relative ranking of DESs, with ChCl/G being the most efficient and ChCl/BD the least efficient, while the fruit extract showed a noticeable difference. Similarly, there was a significant difference in extraction efficiency for CFA between branches and fruits. This is most likely due to differences in matrix composition and the resulting matrix effects. When comparing the results, it is evident that the most efficient DES for each of the three mulberry samples was prepared using a polyalcohol as the HBD. The finding that ChCl/G was the most effective DES tested is consistent with a previous study that examined related analytes and DESs in the analysis of white mulberry leaf extracts using MAE [24]. Another study, which also investigated related phenolic compounds in leaves extracted using UAE, reported that an even more effective DES than the tested ChCl/G was one based on ChCl and citric acid in a molar ratio of 2:1 [23].

#### 2.4.2. Results of TPC Analysis

**Figure 4:**
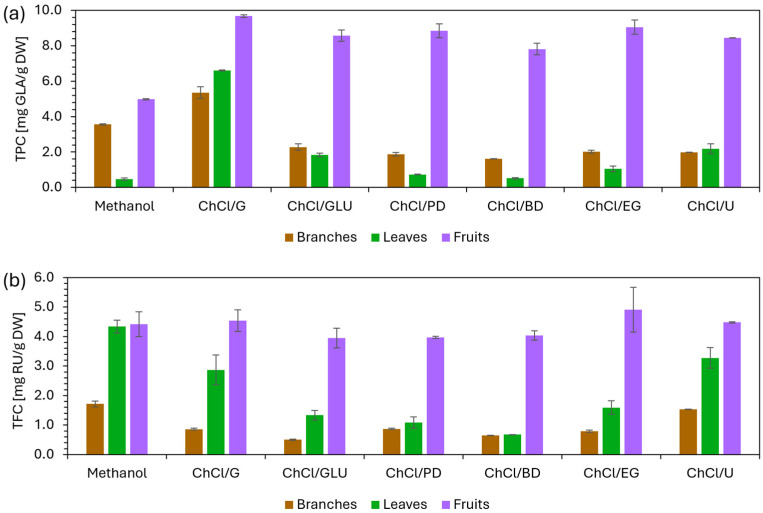
Results of (**a**) TPC and (**b**) TFC analyses for white mulberry branch, leaf, and fruit extracts using different extraction solvents.

The TPC results (Figure 4) show that the most effective solvent for extracting total phenolic compounds was ChCl/G, which achieved the highest values for all three samples. In every case, the yield obtained with ChCl/G exceeded that obtained with MeOH: in the branch extract, it was 1.5 times higher; in the leaf extract, as much as 14 times higher; and in the fruit extract, approximately twice as high. This was the only DES that outperformed methanol for the branch sample, whereas for the leaf and fruit samples, all tested DESs were more effective than the conventional organic solvent tested.

It was expected that the most effective DESs for extracting phenolic compounds would be one of those based on polyalcohols, since their polarity is similar to that of phenolic compounds [24]. In general, higher extraction efficiency can be attributed to interactions between the phenolic compounds and DES components, primarily through hydrogen bonding [39]. Among the polyalcohols used as HBDs, glycerol was the most successful, possessing one more hydroxyl (OH) group than the other polyalcohols tested and thus capable of forming more hydrogen bonds. Although glucose contains two additional OH groups compared to glycerol, the corresponding DES was likely less effective due to its higher viscosity (11.94 mPa·s vs. 7.034 mPa·s at 60 °C). For the remaining three polyalcohol-based DESs, a consistent efficiency trend was observed across all three samples: ChCl/EG > ChCl/PD > ChCl/BD. All three contain the same number of OH groups but differ in the length of their carbon chains. This can be explained by the reduction in polarity and increase in viscosity associated with longer carbon chains (3.993 mPa·s vs. 5.331 mPa·s vs. 6.540 mPa·s at 60 °C), both of which contribute to diminished extraction performance [18].

#### 2.4.3. Results of TFC Analysis

The most effective solvent for extracting total flavonoids, a subgroup of polyphenols, varied slightly among the different sample types. For branches and leaves, methanol was the most efficient solvent, while among the DESs, the urea-based system was the most effective. The ChCl/U yield for branch extract was 10% lower, and 23% lower for leaf extract, than that of methanol. The fruit sample was the only case in which three DESs outperformed the tested organic solvent. The most efficient was ChCl/EG, which achieved an 11% higher yield than methanol. The results of the TFC analysis are shown in Figure 4.

In the extraction of flavonoids using DESs, intermolecular interactions, namely hydrogen bonding and van der Waals forces, play a key role because stronger and more favorable solvent–solute interactions facilitate dissolution and enhance extraction efficiency. Which interactions will be dominant depends on the hydrophobicity of each flavonoid. In addition, steric repulsive forces arising from the increased viscosity of DESs also play an important role, as insufficient mobility limits mass transfer [18,34]. Across all three samples, the most efficient DESs were those based on urea, ethylene glycol, and glycerol as HBDs. In addition to ChCl/U and ChCl/EG having the lowest viscosities, these three HBDs offer the greatest number of hydrogen-bonding functional groups per carbon chain length, aside from glucose. Conversely, the least efficient solvents in all samples were the DESs based on 1,4-butanediol and glucose. The reduced efficiency was likely due to higher viscosity, steric hindrance from larger HBD molecules, and unfavorable polarity. ChCl/GLU is the most viscous among the tested DESs and is also more polar, as the general order of polarity is organic acid-based DESs > sugar-based DESs > polyalcohol-based DESs [20,32]. On the other hand, ChCl/BD is the least polar among the tested polyalcohol-based DESs, having the longest carbon chain. Its molecules also contain the lowest number of OH groups per carbon chain length, resulting in higher viscosity and fewer interactions. Overall, optimal flavonoid extraction requires selecting an HBD with appropriate polarity and balancing hydrogen-bonding groups and carbon chain length to achieve lower viscosity and strong interactions.

## 3. Materials and Methods

### 3.1. Materials and Chemicals

Branch samples of white mulberry (*Morus alba*) were kindly provided by the Faculty of Agriculture and Life Sciences, University of Maribor, Slovenia, from the spring 2023 pruning of their mulberry collection. The branch samples were ground using a variable-speed rotor mill (PULVERISETTE 14 classic line, FRITSCH, Weimar, Germany).

Leaf and fruit samples were freshly collected from a local garden near Maribor, Slovenia, in June 2023, dried at 40 °C for 66 h, and then ground in a Bosch TSM6A011W rotating blade grinder (Gerlingen, Germany). All samples were stored in closed containers kept in a dark environment at −20 °C until further use.

HPLC-grade methanol and HPLC-grade acetonitrile were purchased from J. T. Baker (Radnor, PA, USA). 1,3-Propanediol (98%), urea (≥99%), α-D-glucose (anhydrous, 96%), Folin–Ciocalteu reagent, caffeic acid (≥98.0%), rutin trihydrate (analytical standard), and resveratrol (analytical standard) were obtained from Sigma Aldrich (St. Louis, MO, USA), as well as p-coumaric acid (≥98.0%) from Fluka, Sigma Aldrich (St. Louis, MO, USA). Gallic acid (anhydrous, for synthesis), 1,4-butanediol (99.0%), and acetic acid (glacial, 100%) were bought from Merck (Darmstadt, Germany). Glycerol (99.0–101.0%) and ethylene glycol (≥99.5%) were supplied by Honeywell (Charlotte, NC, USA). Choline chloride (99%) was purchased from Thermo Scientific (Waltham, MA, USA). Phosphoric acid (85%) was bought from Carlo Erba Reagents (Cornaredo, Milan, Italy). Sodium carbonate (anhydrous) was purchased from PENTA Chemicals (Prague, Czech Republic). Aluminum chloride hexahydrate (99%) was obtained from Acros Organics (Geel, Belgium), and sodium acetate (anhydrous) from Gram-Mol (Zagreb, Croatia). Ultrapure water was obtained from a Merck Millipore Milli-Q water purification system (Darmstadt, Germany).

### 3.2. Preparation of DESs

All DESs were prepared using the standard heating and mixing method. Choline chloride was used as the HBA, and six different HBDs, including glycerol, ethylene glycol, 1,3-propanediol, 1,4-butanediol, urea, and glucose, were weighed at the molar ratios listed in Table 3. The mixtures were stirred in sealed bottles placed in a water bath at 80 °C until a homogeneous, colorless liquid formed. After the DESs cooled to room temperature, they were diluted with water at a DES/water ratio of 4/1 (*w*/*w*). The final prepared DESs for extraction efficiency assessment, therefore, contained 20% added water (*w*/*w*).

The establishment of extraction conditions was carried out using DESs based on ChCl and glycerol. For this purpose, DESs containing ChCl and glycerol were prepared in different molar ratios (2:1, 1:1, 1:2, 1:3) with 20% water, as well as in a 1:2 molar ratio and diluted with different amounts of water (10%, 20%, 30%, and 40% *w*/*w*).

### 3.3. Preparation of Standard Solutions

To prepare standard stock solutions (1000 mg/L) of the selected target analytes, 10 mg of each standard was accurately weighed into a separate 10 mL volumetric flask and dissolved in methanol. Working solutions containing all selected analytes at concentrations ranging from 0.5 mg/L to 25 mg/L for GLA, CFA, pCA, and RES, and from 1 mg/L to 25 mg/L for RU due to its weaker signal, were freshly prepared every 3 days by diluting the standard stock solutions with methanol. All solutions were stored at 5 °C when not in use.

### 3.4. Extraction Procedure

Bioactive compounds were extracted from white mulberry samples using the UAE method. A 0.25 g sample (branches, leaves, or fruits) was weighed into a 15 mL conical centrifuge tube, and 5 mL of the selected solvent (methanol or DES) was added. The tightly closed tube was placed in an Iskra Pio Sonis 4 GT ultrasonic cleaning bath (Šentjernej, Slovenia) and sonicated for 30 min at 80 °C (30 kHz, 400 W). Temperature was controlled using the bath’s internal controller, and an external thermometer was used for independent temperature monitoring. After extraction, the mixture was centrifuged for 10 min at 4000 rpm in a Domel Centric 350 centrifuge (Železniki, Slovenia). The supernatant was transferred to a volumetric flask and diluted with water to exactly 5 mL. The diluted extract was filtered through a 0.45 μm nylon membrane filter and stored in a 15 mL closed conical centrifuge tube at 5 °C until further analysis. Extractions were performed in duplicate to assess extraction efficiency and as single extractions to establish extraction conditions.

### 3.5. HPLC-DAD Analysis

A HPLC method was developed for analyzing polyphenolic bioactive compounds in white mulberry samples. The analysis was performed using an HPLC-DAD system (binary pump, series 1100; autosampler, series 1200; column oven, series 1260 Infinity; DAD, series 1200; Agilent Technologies, Santa Clara, CA, USA) with a Gemini C18 column, 150 × 4.60 mm, particle size 5 μm (Phenomenex, Torrance, CA, USA). The injection volume was 15 μL, and the flow rate was 1.1 mL/min. Separation was carried out at room temperature. The detection wavelength was set at 280 nm. The mobile phase consisted of 0.1% aqueous phosphoric acid solution (solvent A) and 100% acetonitrile (solvent B). The separation gradient was as follows: 5–10% B (0–1 min), 10% B (1–3 min), 10–13% B (3–18 min), 13–18% B (18–27 min), 18–23% B (27–34 min), 23–35% B (34–44 min), 35–60% B (44–49 min), 60% B (49–54 min), 60–5% B (54–54.1 min), and 5% B (54.1–60 min). Selected phenolic compounds were identified by comparing their retention times with those of standard compounds under the same chromatographic conditions. Standard calibration curves were used for quantification. Concentrations of target analytes were expressed as mg of analyte per 100 g of dry weight (mg/100 g DW). Differences in analyte concentrations among extraction solvents were assessed using one-way ANOVA followed by Tukey’s HSD test (*p* < 0.05), conducted separately for each plant sample.

### 3.6. Validation of HPLC-DAD Analysis

The developed HPLC method was validated prior to use by assessing the following parameters: linearity, intra- and inter-day precision, LOD, LOQ, accuracy, and selectivity.

Linearity was evaluated over a concentration range of 0.5 mg/L to 25 mg/L for GLA, CFA, pCA, and RES, and from 1 mg/L to 25 mg/L for RU. Calibration curves were prepared using working standard solutions at five levels for RU and six levels for GLA, CFA, pCA, and RES. The correlation coefficient (R^2^) was calculated using the least squares method. Intra-day precision was determined by six consecutive injections of standard solutions at three different concentration levels on the same day. Inter-day precision was determined over three consecutive days by injecting the same three standard solutions once each day. In both cases, precision was expressed as %RSD. LOD and LOQ values were determined from linear calibration curves, using the standard deviation of the y-intercepts of the regression lines as the estimate of the standard deviation of the instrument response, and the value of the calibration curve slopes [40]. Accuracy was assessed by injecting standard solutions, prepared in triplicate at each of three different concentration levels (a total of nine solutions), on the same day. Accuracy was reported as recovery, expressed in percent. Selectivity was partially evaluated by determining whether the extraction solvents interfered with the chromatographic assay. HPLC analysis was performed for blank samples (methanol and six DESs). The presence of potential peaks at the retention times of the target analytes was evaluated.

### 3.7. Determination of Total Phenolic Content (TPC)

The total phenolic content of the extracts was determined using the standard spectrophotometric Folin–Ciocalteu method as described by Razboršek et al. [39], with proportionally adjusted volumes. To 100 µL of prepared extract, 7.9 mL of water and 500 µL of a 10% aqueous solution of Folin–Ciocalteu reagent were added and mixed. After 8 min, 1.5 mL of sodium carbonate solution (200 g/L) was added and mixed. The prepared solutions were kept in the dark for 2 h at room temperature. Standard solutions of gallic acid, ranging from 10 to 500 mg/L, were prepared in methanol. The absorbance of the standards and samples was measured against the blank (methanol) at 765 nm using a UV-Vis spectrophotometer (Agilent 8453, Agilent Technologies, Santa Clara, CA, USA) in triplicate. TPC was expressed as milligrams of gallic acid per gram of dry weight (mg GLA/g DW).

### 3.8. Determination of Total Flavonoid Content (TFC)

The total flavonoid content of the extracts was determined by the standard spectrophotometric method [39], with the volumes proportionally adjusted. To 500 µL of the prepared extract, properly diluted with water if necessary, 1.5 mL of methanol was added and mixed. Then, 0.1 mL of 10% aluminum chloride (in methanol, *w*/*v*), 0.1 mL of 1 M sodium acetate, and 2.8 mL of water were added. The prepared solutions were kept in the dark for 30 min at room temperature. Standard solutions of rutin, with concentrations ranging from 10 to 100 mg/L, were prepared in methanol. The absorbance of the standards and samples was measured against the blank (methanol) at 415 nm using a UV-Vis spectrophotometer (Agilent 8453, Agilent Technologies, Santa Clara, CA, USA) in triplicate. Total flavonoid content was expressed as milligrams of rutin per gram of dry weight (mg RU/g DW).

### 3.9. Measurements of Physicochemical Properties of DES

Density and dynamic viscosity were measured once for all prepared DESs over the temperature range of 10 °C to 60 °C (283.15–333.15 K) in 10 °C increments, as well as at 25 °C. The ambient pressure was 0.1 MPa. An Anton Paar DSA 5000 M instrument (Graz, Austria) was used for density measurements. According to the technical data provided by Anton Paar, the temperature accuracy is ±0.01 °C, the density accuracy is ±7 × 10^−6^ g/cm^3^, and the repeatability of measurements is ±1 × 10^−6^ g/cm^3^. Dynamic viscosities were measured with an Anton Paar Lovis 2000 M rolling-ball viscometer (Graz, Austria), using a capillary with a diameter of 1.59 mm to obtain optimal run times for all prepared DESs, except for ChCl/GLU at 10 °C and ChCl/G 10% from 10 to 20 °C, where a capillary with a diameter of 1.80 mm was used due to higher viscosities. Steel balls were used for all measurements. According to the technical data provided by Anton Paar, viscosity measurements have repeatability up to ±0.1% and accuracy of up to ±0.5%.

## 4. Conclusions

In this study, six choline chloride-based DESs, formulated with hydrogen bond donors from three structural groups (polyalcohols, amides, and sugars), were evaluated for their efficiency in extracting bioactive compounds from three parts of white mulberry (branches, leaves, and fruits). Their performance was compared with conventional methanol extraction. Overall, under the applied extraction conditions, DESs containing polyalcohols as HBDs demonstrated the highest extraction efficiencies, with ChCl/G outperforming other formulations in most cases. Although DESs exhibited lower efficiencies for flavonoid extraction, at least one DES achieved comparable or superior performance to methanol in most tested scenarios. These findings highlight DES as a promising green alternative for extracting polyphenolic bioactive compounds from plant materials such as white mulberry, while acknowledging that the extraction conditions used were standardized and not fully optimized. They provide a foundation for further optimization of each DES to maximize efficiency and tailor the process to specific samples and target compounds.

## Figures and Tables

**Table 1:** Validation parameters for the HPLC method for all target analytes, including retention time (*t*_R_), correlation coefficient (R^2^), intra-day and inter-day precision expressed as relative standard deviation in percentage (%RSD), limit of detection (LOD), limit of quantification (LOQ), and accuracy expressed as percentage recovery.

Validation Parameter		GLA	CFA	pCA	RU	RES
*t*_R_ [min]		3.65 ± 0.03	11.3 ± 0.1	18.1 ± 0.1	29.9 ± 0.3	37.4 ± 0.2
R^2^		0.9993	0.9999	0.9996	0.9999	0.9999
Intra-day precision [%RSD]	1 mg/L	1.42	1.05	1.03	1.35	1.00
	13 mg/L	1.42	0.82	0.28	1.21	0.36
	25 mg/L	1.24	0.62	0.52	0.61	0.47
Inter-day precision [%RSD]	1 mg/L	1.01	1.28	2.6	2.11	1.33
	13 mg/L	2.37	0.6	2.64	1.82	1.21
	25 mg/L	1.00	1.05	0.96	0.91	1.57
LOD [mg/L]		0.6	0.2	0.4	0.3	0.3
LOQ [mg/L]		1.8	0.6	1.4	1.1	0.9
Accuracy [%]	3 mg/L	97 ± 2	100.6 ± 0.6	99 ± 1	102 ± 3	98 ± 2
	13 mg/L	98 ± 3	102.4 ± 0.3	101 ± 1	101 ± 3	103 ± 2
	23 mg/L	100 ± 1	100.7 ± 0.9	98 ± 2	99 ± 3	100.4 ± 0.6

**Table 2:** Results of HPLC analysis of white mulberry branch, leaf, and fruit extracts using different extraction solvents, expressed as mg/100 g DW.

Sample	Solvent	GLA	CFA	pCA	RU	RES
Branches	Methanol	/	5.0 ± 0.1 ^a^	<LOQ	6.3 ± 0.1 ^a^	<LOQ
	ChCl/G	/	5.1 ± 0.3 ^a^	<LOQ	4.0 ± 0.3 ^b^	<LOQ
	ChCl/GLU	/	5.1 ± 0.2 ^a^	<LOQ	3.6 ± 0.4 ^b,c^	<LOQ
	ChCl/PD	/	4.4 ± 0.2 ^a^	<LOQ	3.40 ± 0.08 ^b,c^	<LOQ
	ChCl/BD	/	3.4 ± 0.1 ^b^	<LOQ	2.8 ± 0.2 ^c^	<LOQ
	ChCl/EG	/	4.7 ± 0.3 ^a^	<LOQ	3.5 ± 0.1 ^b,c^	<LOQ
	ChCl/U	/	4.8 ± 0.1 ^a^	<LOQ	3.4 ± 0.1 ^b,c^	<LOQ
Leaves	Methanol	<LOQ	/	<LOD	4.5 ± 0.4 ^c,d^	<LOQ
	ChCl/G	<LOQ	/	<LOD	8.4 ± 0.4 ^a^	<LOQ
	ChCl/GLU	<LOQ	/	<LOD	6.6 ± 0.8 ^b^	<LOQ
	ChCl/PD	<LOQ	/	<LOD	3.6 ± 0.2 ^d^	<LOQ
	ChCl/BD	<LOQ	/	/	3.39 ± 0.05 ^d^	<LOQ
	ChCl/EG	<LOQ	/	<LOD	5.1 ± 0.2 ^c^	<LOQ
	ChCl/U	<LOQ	/	<LOD	5.51 ± 0.06 ^b,c^	<LOQ
Fruits	Methanol	NQ	11.7 ± 0.2 ^d^	/	27.3 ± 0.3 ^a^	<LOQ
	ChCl/G	NQ	14.1 ± 0.1 ^c^	/	20.6 ± 0.8 ^b^	/
	ChCl/GLU	NQ	14.7 ± 0.2 ^c^	/	18.76 ± 0.09 ^b^	/
	ChCl/PD	NQ	18 ± 1 ^b^	<LOQ	23 ± 2 ^a,b^	<LOQ
	ChCl/BD	NQ	11.4 ± 0.4 ^d^	<LOD	20 ± 1 ^b^	<LOQ
	ChCl/EG	NQ	21.6 ± 0.4 ^a^	<LOD	21.9 ± 0.1 ^b^	<LOQ
	ChCl/U	NQ	15.4 ± 0.5 ^c^	/	19 ± 1 ^b^	<LOQ

/—no peak detected; not quantified (NQ)—peak not quantified due to insufficient separation; <LOD—concentration below the limit of detection; <LOQ—concentration below the limit of quantification; ^a^, ^b^, ^c^, ^d^—different letters indicate significant differences among means for each quantified analyte, separately for each sample type (one-way ANOVA followed by Tukey’s HSD test, *p* < 0.05).

**Table 3:** Composition and molar ratio of the prepared DESs.

Abbreviation	Composition (HBA/HBD)	Molar Ratio
ChCl/G	Choline chloride/glycerol	1:2
ChCl/EG	Choline chloride/ethylene glycol	1:2
ChCl/PD	Choline chloride/1,3-propanediol	1:2
ChCl/BD	Choline chloride/1,4-butanediol	1:2
ChCl/U	Choline chloride/urea	1:2
ChCl/GLU	Choline chloride/glucose/water	2:1:1

## Data Availability

Data are contained within the article and Supplementary Materials.

## Supplementary Materials

The following supporting information can be downloaded at https://www.mdpi.com/article/10.3390/molecules31071193/s1: Figure S1: HPLC chromatogram of mulberry branch extract prepared with methanol as the solvent; Figure S2: HPLC chromatogram of mulberry branch extract prepared with ChCl/G as the most efficient DES solvent; Figure S3: HPLC chromatogram of mulberry leaf extract prepared with methanol as solvent; Figure S4: HPLC chromatogram of mulberry leaf extract prepared with ChCl/G as the most efficient DES solvent; Figure S5: HPLC chromatogram of mulberry fruit extract prepared with methanol as solvent; Figure S6: HPLC chromatogram of mulberry fruit extract prepared with ChCl/EG as the most efficient DES solvent; Table S1: Densities of prepared DESs in the temperature range from 10 to 60 °C; Table S2: Viscosities of prepared DESs in the temperature range from 10 to 60 °C.

## Abbreviations

The following abbreviations are used in this manuscript:

CFA	Caffeic acid
ChCl	Choline chloride
DAD	Diode array detector
DES	Deep eutectic solvent
DW	Dry weight
GLA	Gallic acid
HBA	Hydrogen bond acceptor
HBD	Hydrogen bond donor
LOD	Limit of detection
LOQ	Limit of quantification
MAE	Microwave-assisted extraction
NQ	Not quantified
OH	Hydroxyl group
pCA	p-coumaric acid
RES	Resveratrol
%RSD	Relative standard deviation
RU	Rutin
TFC	Total flavonoid content
TPC	Total phenol content
UAE	Ultrasound-assisted extraction
