# Supplementary material for: Choline Chloride-Based Deep Eutectic Solvents for Efficient Polyphenol Extraction from White Mulberry (Morus alba)
Source: Molecules. 2026 Apr 3;31(7):1193. doi: 10.3390/molecules31071193 (PMC13074552; doi:10.3390/molecules31071193)
Supplement: Supplementary file 1 [file molecules-31-01193-s001.zip › molecules-4137729-supplementary.pdf]

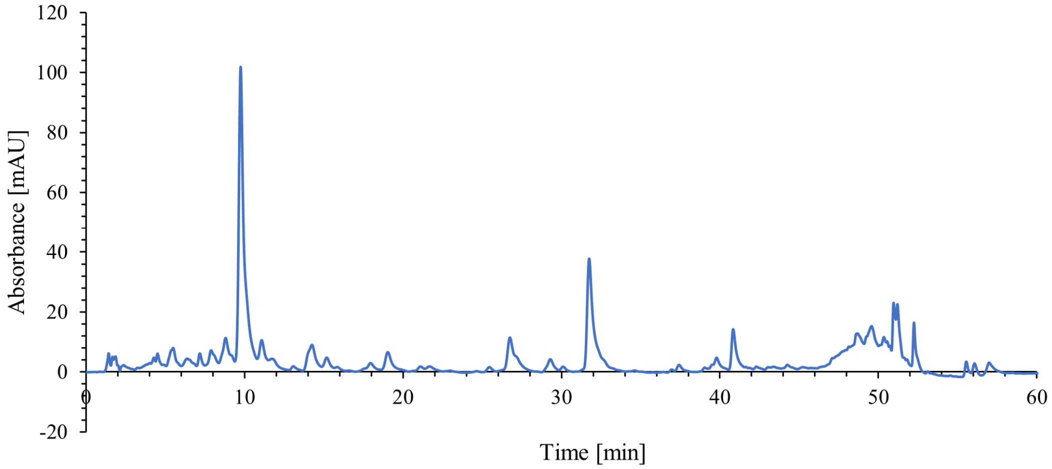

Figure S1. HPLC chromatogram of mulberry branch extract prepared with methanol as the solvent.

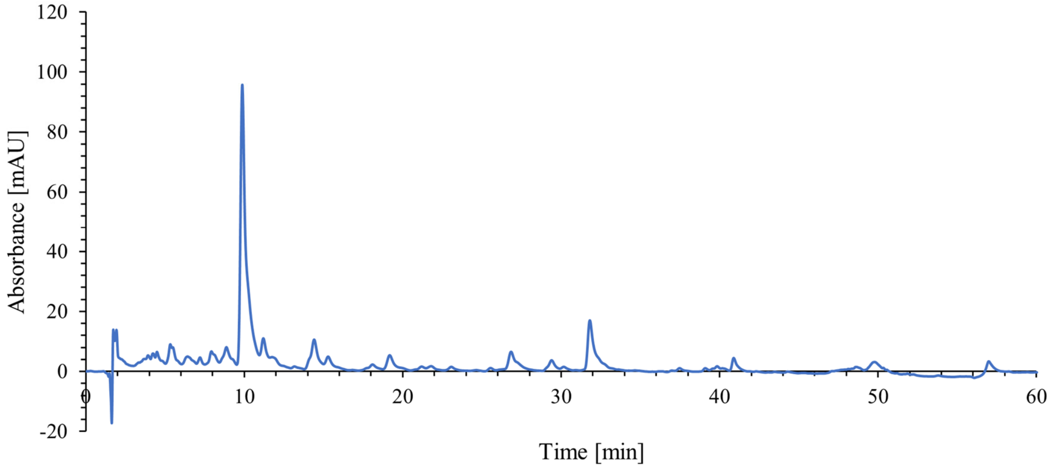

Figure S2. HPLC chromatogram of mulberry branch extract prepared with ChCl/G as the most efficient DES solvent.

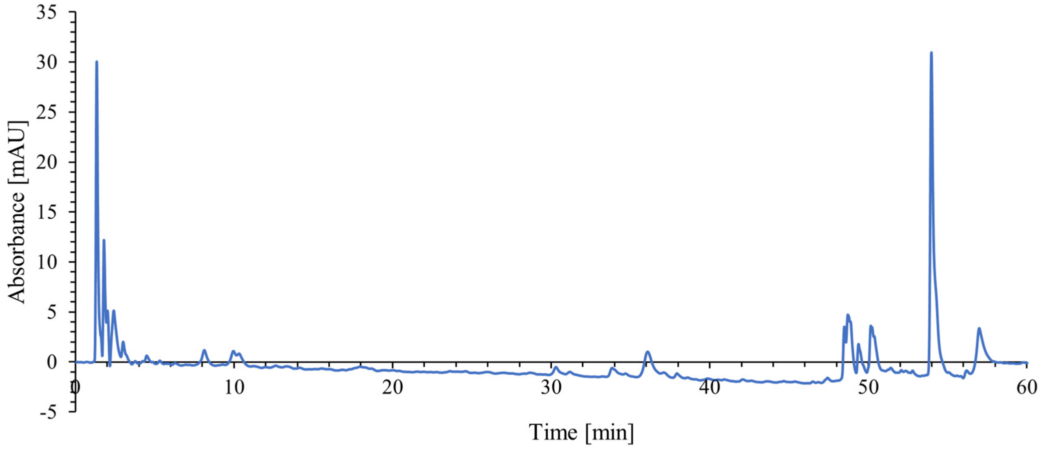

Figure S3. HPLC chromatogram of mulberry leaf extract prepared with methanol as solvent.

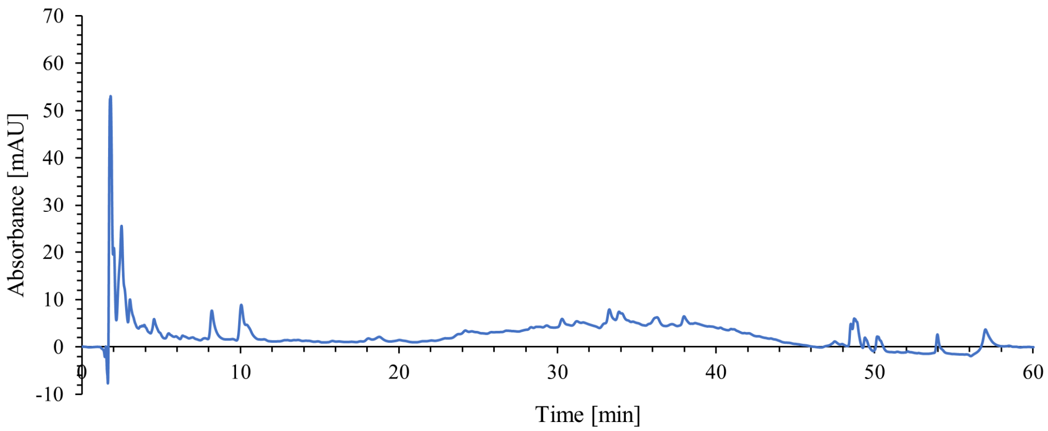

Figure S4. HPLC chromatogram of mulberry leaf extract prepared with ChCl/G as the most efficient DES solvent.

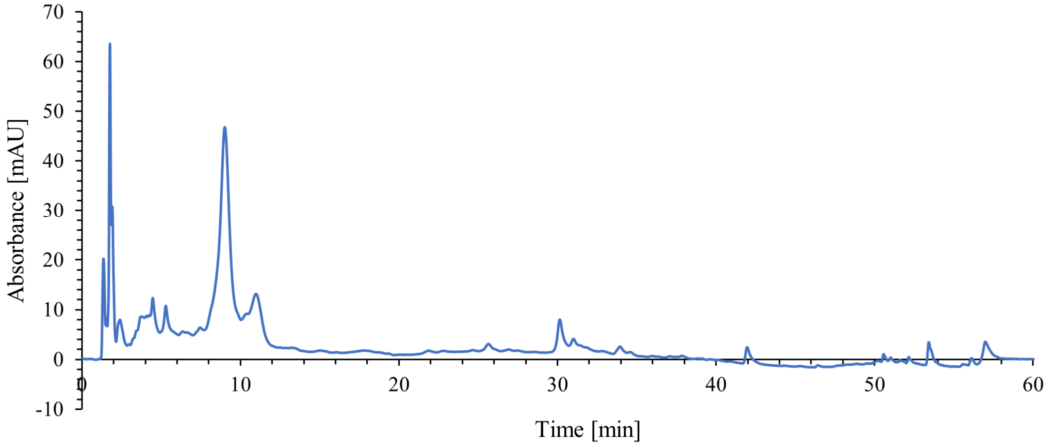

Figure S5. HPLC chromatogram of mulberry fruit extract prepared with methanol as solvent.

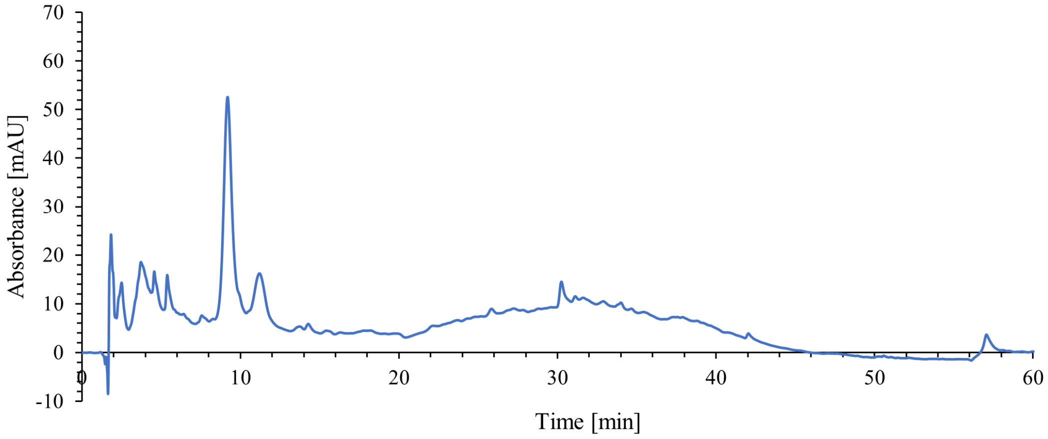

Figure S6. HPLC chromatogram of mulberry fruit extract prepared with ChCl/EG as the most efficient DES solvent.

Table S1. Densities of prepared DESs in the temperature range from 10 to 60 °C.

DES	Density [g/cm^3]						
	**9.998 °C**	**19.998 °C**	**24.998 °C**	**29.998 °C**	**39.998 °C**	**49.998 °C**	**60.002 °C**
ChCl/EG	1.105744	1.100254	1.097494	1.094720	1.089139	1.083505	1.077798
ChCl/PD	1.081380	1.075515	1.072554	1.069576	1.063553	1.057453	1.051268
ChCl/BD	1.065463	1.059955	1.057179	1.054391	1.048763	1.043062	1.037290
ChCl/U	1.161510	1.156204	1.153547	1.150885	1.145540	1.140170	1.134761
ChCl/GLU	0.196648	0.193505	0.192416	0.191595	0.190645	0.190509	0.191084
ChCl/G 2:1	1.129712	1.124797	1.122330	1.119849	1.114866	1.109837	1.104769
ChCl/G 1:1	1.143726	1.138630	1.136069	1.133493	1.128301	1.123043	1.117734
ChCl/G 1:2 20%	1.161789	1.156472	1.153793	1.151091	1.145628	1.140082	1.134457
ChCl/G 1:3	1.172581	1.167126	1.164376	1.161597	1.155977	1.150254	1.144436
ChCl/G 10%	1.180893	1.175392	1.172644	1.169894	1.164388	1.158834	1.153244
ChCl/G 30%	1.141706	1.136592	1.133994	1.131364	1.126012	1.120535	1.114919
ChCl/G 40%	1.121057	1.116240	1.113767	1.111249	1.106067	1.100693	1.095121

Table S2. Viscosities of prepared DESs in the temperature range from 10 to 60 °C.

DES	Viscosity [mPa·s]						
	**10.00 °C**	**20.00 °C**	**25.00 °C**	**30.00 °C**	**40.00 °C**	**50.00 °C**	**60.00 °C**
ChCl/EG	18.63	12.60	10.56	8.947	6.617	5.055	3.993
ChCl/PD	36.39	22.18	17.77	14.44	9.926	7.138	5.331
ChCl/BD	43.68	27.05	21.77	17.75	12.24	8.792	6.540
ChCl/U	19.24	12.98	10.89	9.263	6.893	5.337	4.267
ChCl/GLU	109.6	62.58	46.77	36.96	24.15	16.62	11.94
ChCl/G 2:1	46.11	29.39	23.98	19.84	14.05	10.35	7.883
ChCl/G 1:1	45.49	28.32	22.90	18.78	13.10	9.542	7.203
ChCl/G 1:2 20%	50.88	30.47	24.22	19.56	13.30	9.482	7.034
ChCl/G 1:3	56.73	33.05	25.97	20.76	13.87	9.741	7.136
ChCl/G 10%	173.7	93.76	69.26	54.43	33.70	22.48	15.75
ChCl/G 30%	20.49	13.28	10.94	9.124	6.567	4.916	3.810
ChCl/G 40%	10.19	6.984	5.896	5.033	3.780	2.931	2.336
